# Syndrome of inappropriate antidiuretic hormone secretion associated with an ovarian immature teratoma: A case report and review of the literature

**DOI:** 10.1016/j.gore.2021.100910

**Published:** 2021-12-16

**Authors:** Marianne Hom-Tedla, Allison Brodsky, Oluwole Fadare, Claire Meriwether, Michael McHale

**Affiliations:** a Department of Gynecologic Oncology, University of California, San Diego, San Diego, CA, United States; b Department of Pathology, University of California, San Diego, San Diego, CA, United States

**Keywords:** Hyponatremia, Immature teratoma, Surgery

## Abstract

• SIADH can be associated with an ovarian immature teratoma.
• Medical management of SIADH improved but did not resolve the hyponatremia.
• Complete resolution of the hyponatremia was only obtained after surgical debulking.

## 1 Introduction

Syndrome of inappropriate antidiuretic hormone secretion (SIADH) causes hyponatremia through increased secretion of vasopressin. Paraneoplastic SIADH has been well documented with small cell carcinoma of the lung. The following case report describes refractory hyponatremia due to SIADH associated with an immature teratoma. Complete resolution occurred following surgical debulking.

## 2 Case

A 27-year-old previously healthy gravida 1, para 1, presented with a three month history of worsening diffuse abdominal discomfort, nausea, and vomiting. She also reported a one week of fatigue, dizziness, blurred vision, and a mild headache. She denied syncope, seizure-like activity, urinary changes, and abnormal vaginal bleeding. Her exam was notable for a large abdominopelvic mass and no clinical signs of fluid overload nor edema. On contrast-enhanced CT scan, there was a 20-centimeter complex mass with associated ascites, peritoneal thickening, omental infiltration, and borderline retroperitoneal lymphadenopathy. Tumor markers were elevated with a CA-125 of 191 U/mL and an AFP of 69.3 ng/mL. Her LDH was normal at 150 U/L and her quantative HCG was <1 mIU/mL. On pre-operative labs, she was found to have a serum sodium of 116 mmol/L. Her liver enzymes and her other electrolytes were within normal limits.

She was admitted for further hyponatremia work-up and management. Orthostatic blood pressures were obtained and were consistent with euvolemia. Admission labs were consistent with SIADH with the following significant results: urine chloride was 162 mmol/L, urine sodium was 229 mmol/L, and urine osmolality was 891 mOsm/kg. Her thyroid stimulating hormone and morning cortisol were within normal limits. Upon admission, she initially received intravenous fluid resuscitation due to concerns for diarrhea associated hypovolemic hyponatremia. This intervention worsened her hyponatremia, further suggesting the diagnosis of SIADH. As such, her management then included strict fluid restriction to 1.5 L daily, 1 g salt tabs three times daily, and intermittent furosemide infusions. Her salt tabs were increased to 2 g three times daily and her furosemide was changed to scheduled 20 mg twice daily after minimal improvement. After 5 days there was a modest improvement of the serum sodium but hyponatremia persisted.

During this admission, she became febrile to 101.9 degrees Fahrenheit. A full sepsis work-up was negative aside from the detection of enteropathogenic E. Coli (EPEC) on stool testing. This finding was consistent with her reported history of intermittent diarrhea, and she was treated with a 7-day course of antibiotics.

She ultimately underwent an exploratory laparotomy with resection of the 20-centimeter tumor involving the right ovary and fallopian tube. Intraoperative findings also included 3 L of ascites with multiple 2 mm nodules in the omentum and pelvic cul-du-sac, which were removed. She was repleted with 500 ml of albumin and 1 L of normal saline. The intraoperative rapid frozen section pathologic evaluation of the tumor was inconclusive, and given the patient’s desire for preservation of fertility, the decision was made to resect the peritoneal nodules and omentum. There were no complications from her surgery.

**Fig. 1:**
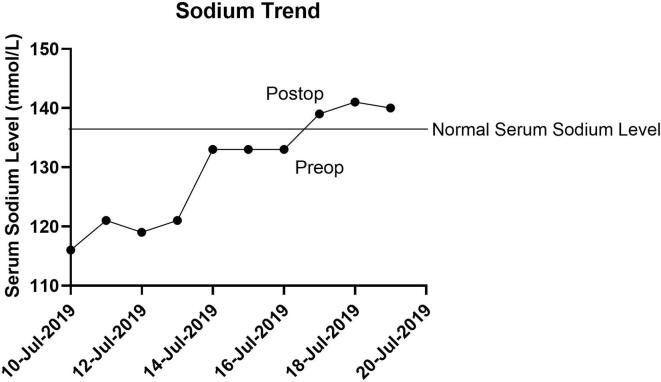
Serum Sodium Trend.

The final pathology demonstrated stage 3B grade 2 immature teratoma arising from the right ovary. On postoperative day 1, her serum sodium normalized and remained within normal limits throughout her hospitalization as demonstrated in Fig. 1. Post-operatively she received four cycles of bleomycin, etoposide, and carboplatin, with no further hyponatremia encountered. She had a complete response to the chemotherapy, which was confirmed with a second look laparoscopy. She has remained without evidence of disease and with a normal serum sodium level.

## 3 Pathology

**Fig. 2:**
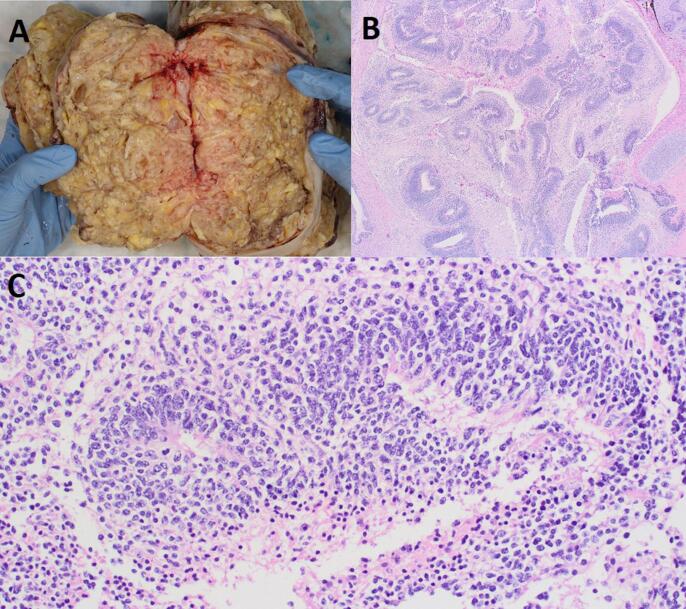
**Pathologic examination.** (A) Macroscopic appearance of the ovarian tumoral cut surface. (B) Low magnification image of an “immature” area of tumor, comprised of abundant immature neuroepithelial elements. (C) High power magnification demonstrated neuroepithelial elements comprised of aggregates of primitive appearing, mitotically active cells forming rosette-like structures.

Pathologic examination of the specimen revealed immature teratoma elements involving the right ovary, omentum, and peritoneal nodules. The ovarian tumor measured 20 cm in greatest dimension and weighed 1704.4 g. The cut section of the bivalved tumor demonstrated an irregular, predominantly solid, sporadically cystic, yellowish mass (Fig. 2A). Microscopic evaluation demonstrated a preponderance (approximately 80%) of mature elements, including cartilage, bone, skin, respiratory and gastrointestinal epithelium, among others. The remaining 20% of the ovarian tumor, as well as the omental and peritoneal lesions, demonstrated immature neuroepithelial elements, comprised of scattered aggregates of primitive appearing, mitotically active cells forming rosette-like structures (Fig. 2B and C). The maximal number of low-power microscopic fields (40x total magnification) showing aggregates of immature neuroepithelium in any one slide of the ovarian tumor was 3, consistent with a grade 2 tumor on a 3-tiered scale and a high-grade tumor on a 2-tiered scale.

## 4 Discussion

This patient presented with classic symptoms of hyponatremia: nausea, headache, and fatigue. Having a normal thyroid stimulating hormone level makes the diagnosis of hypothyroid-related hyponatremia less likely. She was not taking medications at the time of her diagnosis. The patient did have malignant ascites as well as diarrhea, which could lead to hypotonic hyponatremia; however, she did not respond to fluid resuscitation. The patient also had an inappropriately high urine sodium level, which commonly is associated with SIADH. She partially responded to medical management, water restriction, and salt tablets; however, the serum sodium did not normalize until the immature teratoma was resected.

One of the initial cases of refractory hyponatremia was associated with a serous ovarian carcinoma with neuroendocrine differentiation (Taskin et al., 1996). There have since been six published cases of immature teratoma related hyponatremia (Lam and Cheung, 1996, Lam and Yu, 2004, Roman et al., 2004, Sakamoto et al., 2016, Iqbal and Schwenk, 2018, Tejani et al., 2020). Similar to the case described, although the hyponatremia improved with medical management, it did not resolve until surgical debulking in 66% of the cases (Lam and Cheung, 1996, Lam and Yu, 2004, Sakamoto et al., 2016, Iqbal and Schwenk, 2018, Tejani et al., 2020). Roman demonstrated serologically in their case that endogenous serum vasopressin levels were low, supporting evidence of tumor secreting vasopressin (Roman et al., 2004). The majority of the cases were treated with a combination of surgery and chemotherapy. Our patient is one of the first presentations in the United States of immature teratoma related SIADH. A summary of the available cases of immature teratoma associated SIADH is listed in Table 1.

**Table 1:** Summary of related cases.

Author	Year published	Age at diagnosis	Serum Sodium Nadir	Diagnosis	Treatment
Lam and Cheung (1996) (Hong Kong)	1996	17	115	Stage 1C, grade 2, immature teratoma	Surgery, Chemotherapy
Lam and Yu (2004) (Hong Kong)	2004	17	121	Grade 2, immature teratoma	Surgery, Chemotherapy
Roman et al. (2004) (France)	2004	22	111	Stage 3, grade 2 immature teratoma	Surgery, Chemotherapy
Sakamoto et al. (2016) (Japan)	2016	16	119	Stage 1C, grade 2 immature teratoma	Surgery. Chemotherapy
Iqbal and Schwenk (2018) (US)	2018	12	123	Stage 3B, immature teratoma	Surgery
Tejani et al. (2020) (US)	2020	32	116	Stage 1A, grade 2 immature teratoma	Surgery, Chemotherapy

## 5 Conclusion

Malignancy associated syndrome of inappropriate antidiuretic hormone secretion is rare. Here, we describe a case of immature teratoma associated SIADH which was refractory to medical therapy and only resolved following surgical debulking. Hyponatremia due to paraneoplastic syndrome is rare, but should be considered in the diagnosis differential of a young woman with a pelvic mass and refractory hyponatremia.

## 6 Informed consent statement

Informed consent to write the case report was obtained from the patient.

## Author contribution

All of the listed authors in this manuscript were an active part of the surgical and medical treatment of the patient, as well as contributed to the writing of the manuscript.

## Declaration of Competing Interest

The authors declare that they have no known competing financial interests or personal relationships that could have appeared to influence the work reported in this paper.
